# Targeting the Fanconi Anemia Pathway to Identify Tailored Anticancer Therapeutics

**DOI:** 10.1155/2012/481583

**Published:** 2012-05-24

**Authors:** Chelsea Jenkins, Jenny Kan, Maureen E. Hoatlin

**Affiliations:** Department of Biochemistry and Molecular Biology, Oregon Health and Science University, 3181 SW Sam Jackson Parkway, Portland, OR 97239, USA

## Abstract

The Fanconi Anemia (FA) pathway consists of proteins involved in repairing DNA damage, including interstrand cross-links (ICLs). The pathway contains an upstream multiprotein core complex that mediates the monoubiquitylation of the FANCD2 and FANCI heterodimer, and a downstream pathway that converges with a larger network of proteins with roles in homologous recombination and other DNA repair pathways. Selective killing of cancer cells with an intact FA pathway but deficient in certain other DNA repair pathways is an emerging approach to tailored cancer therapy. Inhibiting the FA pathway becomes selectively lethal when certain repair genes are defective, such as the checkpoint kinase ATM. Inhibiting the FA pathway in ATM deficient cells can be achieved with small molecule inhibitors, suggesting that new cancer therapeutics could be developed by identifying FA pathway inhibitors to treat cancers that contain defects that are synthetic lethal with FA.

## 1. Introduction

Fanconi anemia is a rare genetic disease featuring characteristic developmental abnormalities, a progressive pancytopenia, genomic instability, and predisposition to cancer [1, 2]. The FA pathway contains a multiprotein core complex, including at least twelve proteins that are required for the monoubiquitylation of the FANCD2/FANCI protein complex and for other functions that are not well understood [3–6]. The core complex includes the Fanconi proteins FANCA, FANCB, FANCC, FANCE, FANCF, FANCG, FANCL, and FANCM. At least five additional proteins are associated with the FA core complex, including FAAP100, FAAP24, FAAP20, and the histone fold dimer MHF1/MHF2 [1, 4, 7–10]. The core complex proteins function together as an E3 ubiquitin ligase assembly to monoubiquitylate the heterodimeric FANCI/FANCD2 (ID) complex. The monoubiquitylation of FANCD2 is a surrogate marker for the function of the FA pathway [11]. USP1 and its binding partner UAF1 regulate the deubiquitination of FANCD2 [12]. The breast cancer susceptibility and Fanconi proteins FANCD1/BRCA2, the partner of BRCA2 (PALB2/FANCN), a helicase associated with BRCA1 (FANCJ/BACH1), and several newly identified components including FAN1, FANCO/RAD51C, and FANCP/SLX4 [13–17] participate in the pathway to respond to and repair DNA damage (for review, see [5]).

**Figure 1:**
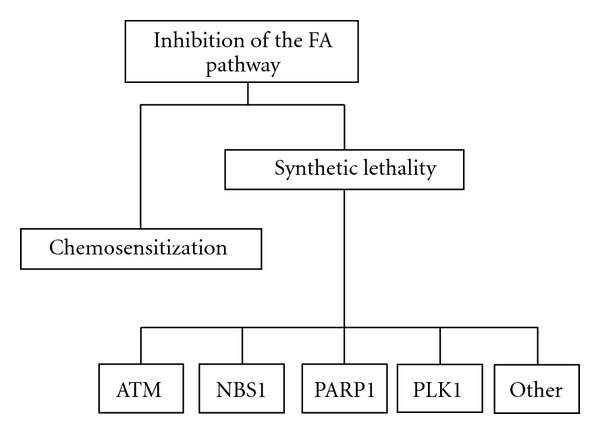
Inhibition of the FA pathway. Strategy for selectively targeting tumor cells by inhibition of the FA pathway by (a) chemosensitization to cross-linking agents or by (b) exploiting specific synthetic lethal interactions.

 Although FA is rare, understanding the functional role of the FA proteins in context with other DNA damage response pathways will provide broader opportunities for new cancer therapeutics. Two general strategies could accomplish this, as illustrated in Figure 1: inhibiting the FA pathway in tumor cells to sensitize them to cross-linking agents, or by exploiting synthetic lethal relationships. The latter approach depends on inhibiting the FA pathway in tumor cells that are defective for a secondary pathway required for survival in the absence of the FA pathway.

## 2. Chemosensitizing and Resensitizing Tumor Cells

A defining characteristic of FA cells is hypersensitivity to cross-linking agents, such as the chemotherapeutic agent cisplatin [2, 5]. Cisplatin (and other platinum-based compounds) has been used as a chemotherapeutic drug for over 30 years (for review see [18]). The toxicity of platinum-based chemotherapy (nephrotoxicity, neurotoxicity, and ototoxicity) and development of cisplatin resistance are limitations of the therapy [18–20]. Once inside the cell, cisplatin enters the nucleus and forms covalent DNA interstrand cross-links via platinum-DNA adducts. These cross-links block ongoing DNA replication, and in the absence of repair, activate apoptotic pathways [18, 19]. A functional FA pathway is required for processing damage after exposure to cisplatin and other crosslinking agents, and is at least partially responsible for resistance to cisplatin. Cell-free and cell-based assays have identified inhibitors of the FA pathway, and some of these inhibitors can resensitize platinum-resistant tumors and cell lines [19, 21, 22]. Further efforts to identify small molecule compounds that specifically inhibit the FA pathway could lead to improved resensitization from treatment-induced resistance. 

## 3. Exploiting Synthetic Lethal Interactions

In addition to sensitization, inhibiting the FA pathway may be an effective strategy to exploit synthetic lethal interactions aimed at improving targeted killing of tumor cells. Current approaches in cancer treatment are generally not selective, affecting both cancer cells and normal cells. However, inactivation of DNA repair pathways, an event that occurs frequently during tumor development [23], can make cancer cells overdependent on a reduced set of DNA repair pathways for survival. There is new evidence that targeting the remaining functional pathways by using a synthetic lethal approach can be useful for single-agent and combination therapies in such tumors. Two genes have a synthetic lethal relationship if mutants for either gene are viable but simultaneous mutations are lethal [20]. A successful example of this approach is specific targeting of BRCA-deficient tumors with PARP (poly (ADP-ribose) polymerase) inhibitors [24].

## 4. Defects in Homologous Recombination and Sensitivity to PARP Inhibitors

Defects in HR repair can result in an overreliance on the protein PARP1, which is responsible for repair of DNA single strand breaks by the base excision repair pathway. Unrepaired single-strand breaks are converted to double-strand breaks during replication and must be repaired by HR [25–27]. Thus, treating cells that are defective in HR with PARP inhibitors results in a targeted killing of the defective cells, while cells with intact HR are capable of repair. Defects in breast cancer susceptibility proteins BRCA1 and BRCA2 (FANCD1) result in HR defects [28]. Clinical trials investigating the effectiveness of PARP inhibitors against recurrent ovarian cancer have been promising, but rigorous stratification of tumors for HR status or “BRCA-ness” (defects in HR) is needed to identify the patients who are likely to benefit [29–31]. Future clinical trials with PARP1 inhibitors in breast cancer may require combination therapies, evaluation of resistance, and identification of non-BRCA biomarkers [32].

PARP1 Inhibition has also been shown to be selectively toxic to ATM-defective tumor cell lines *in vitro* and to increase radiosensitivity of other ATM-proficient cell lines, including nonsmall-cell lung cancer, medulloblastoma, ependymoma, and high-grade gliomas [33–35]. In addition, cell lines lacking functional Mre11 are sensitive to PARP1 inhibitors, strengthening the case for combined use of PARP1 inhibitors with inhibitors of the FA pathway [36, 37].

**Figure 2:**
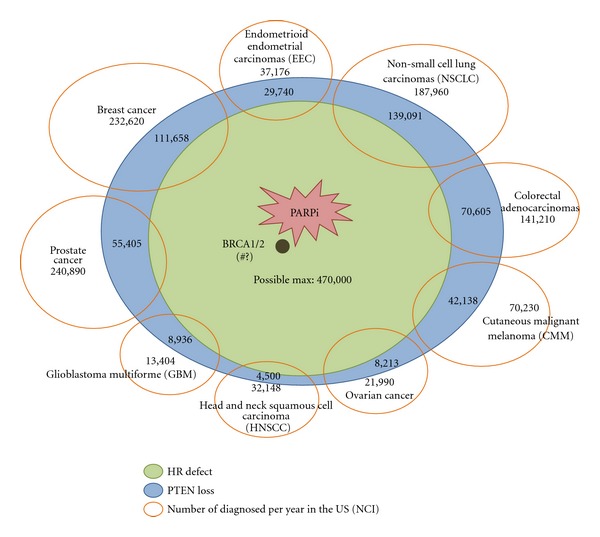
PTEN defects in cancers. Types of cancer diagnosed annually in the US (orange oval), with the estimates for PTEN deficiencies shown in each type (blue oval). An unknown percentage of tumors with PTEN deficiencies will have a defect in homologous recombination (HR) repair, predicting sensitivity to treatment with PARP1 inhibitors (green oval).

PTEN (phosphatase and tensin homolog) is a tumor-suppressor gene and one of the most commonly mutated genes in human tumor cells [38, 39] (see Figure 2). PTEN deficiency results in decreased expression of RAD51, which is required for homologous recombination [38, 40]. PTEN deficient tumors are thus candidates for targeted therapy by PARP1 inhibition [36, 38]. Although approximately 470,000 (48%) of 977,628 newly diagnosed cancers each year in the US may have PTEN defects, only a subset of these cancers will have PTEN mutations that result in homologous recombination defects and sensitivity to PARP inhibitors [28, 39, 41–51]. Current studies are aimed at determining the relationship between PTEN loss, RAD51 expression, and PARP1 inhibitor sensitivity [36]. Efforts to asses HR status to establish which PTEN mutations lead to an HR defect, and determining under what circumstances RAD51 expression could be used as a biomarker, will be useful to stratify and predict PARP1 inhibitor sensitivity.

Synthetic lethal interactions with the FA pathway have been explored. An siRNA-based screen of cells deficient in the Fanconi core complex protein, FANCG, showed that ATM, PARP1, NBS1, and PLK1 were among the genes with a synthetic lethal interaction [52] (see Table 1). The FA-ATM synthetic lethal relationship is particularly interesting since ATM deficiency has been reported in a subset of patients with hematological malignancies, including mantle cell lymphoma, chronic lymphocytic leukemia, and acute lymphoblastic leukemia [53, 54], making these potential targets for treatment with FA pathway inhibitors (see Table 2).

## 5. Inhibiting the FA Pathway

**Figure 3:**
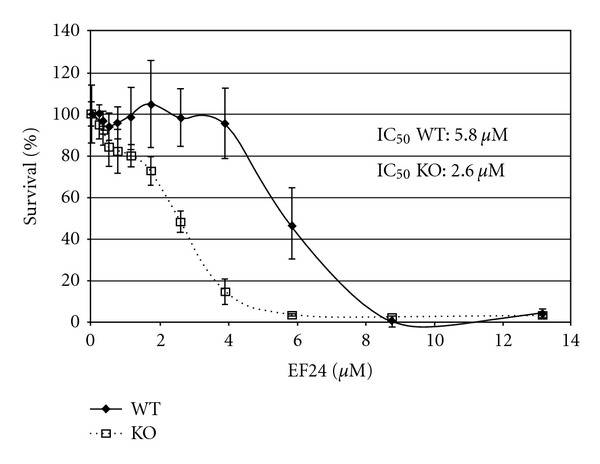
EF24 is selectively toxic to ATM-deficient cells [57]. 309ATM-deficient and 334ATM wild type cells were treated with the FA pathway inhibitor EF24. Cell viability was measured after 3 days by MTS assay. Each point represents the mean of 3 repeats. Error bars represent standard deviation.

Inhibition of the FA pathway could occur at any point in the multistep FA protein network, but a key predictive readout for FA function and resistance to ICLs is the monoubiquitylation of FANCD2 [11, 55]. Several inhibitors of FANCD2 monoubiquitylation have been identified including proteasome inhibitors bortezomib and MG132, curcumin, and the curcumin analogs EF24 and 4H-TTD [19, 22, 56, 57]. Curcumin, a natural product derived from turmeric, was identified as a weak inhibitor of FANCD2 monubiquitylation in a cell-based screen [19]. We developed a cell-free assay in *Xenopus* egg extracts to screen small molecules for stronger and more specific inhibitors of FANCD2 monubiquitylation. Unlike cell-based screening assays for small molecules capable of inhibiting the FA pathway, the cell-free method uncouples FANCD2 monoubiquitylation from DNA replication, thus focusing more specifically on the key biochemical steps in a soluble context enriched for nuclear proteins and capable of full genomic replication [22]. Screening in egg extracts identified 4H-TTD, a compound with structural similarity to curcumin as an inhibitor, and this inhibitory effect was verified in human cells [22, 57]. A series of curcumin analogs were also tested, including EF24, a potent monoketone analog of curcumin [58, 59]. The prediction that an FA inhibitor would selectively kill ATM-deficient cells was tested in cell-based assays for synthetic lethality in ATM-proficient and ATM-deficient cells. ATM-deficient cells treated with EF24 demonstrated an increased sensitivity compared to ATM wt cells (see Figure 3) [22, 57]. The increased lethality in ATM-deficient cells provides evidence for future synthetic lethal approaches with FA pathway inhibitors in the treatment of ATM-deficient tumors, and other tumors with deficiencies in genes that are synthetically lethal with FA (see Table 1) [6, 52]. 

## 6. Conclusion and Future Directions

Understanding how the Fanconi anemia pathway functions in concert with other DNA damage response networks is essential for understanding genomic stability and for exploiting synthetic lethality for new cancer treatments. New chemotherapeutic agents could be developed by identifying potent and specific inhibitors of the FA pathway, for example, by screening for compounds that inhibit key FA pathway steps (e.g., monoubiquitylation and deubiquitylation of FANCD2/FANCI). While a long-term defect in the function of the FA pathway would result in genomic instability, short-term inhibition could provide a treatment strategy for tumors with deficiencies in certain other DNA repair pathways. Stringent identification of additional genes with synthetic lethal relationships with the FA pathway, and identification of malignancies with deficiencies or mutations in genes that are synthetic lethal with FA will be required for these tailored therapeutic approaches.

## Figures and Tables

**Table 1:** Function and expression of genes synthetically lethal with FA.

Gene synthetically lethal with FA genes	Function	Expression in tumor cells
TREX2 [52]	DNA exonuclease; SAGA complex pathway	Expressed in most tumor cell lines [60]
PARP1 [52]	BER	Overexpressed in tumors, including medulloblastoma, ependymoma, HGG, melanoma, and breast cancers [35, 61–63]
PLK1 [52]	Cell-cycle progression	Over-expressed in many human tumors [64]
RAD6/HR6B [52]	Switching of DNA polymerases	Upregulated in metastatic mammary tumors [65]
CDK7 [52]	Transcription	Moderately over-expressed in tumor cell lines [66]
TP53BP1 [52]	DSB sensing; ATM activation	Underexpressed in most cases of triple negative breast cancer [67]
ATM [52]	DSB response kinase	Under-expressed in some tumors, see Figure 3
NEIL1 [52]	BER	Expression reduced in 46% of gastric cancers [68]
RAD54B [52]	HR	Known to be mutated in cancer cell lines [69, 70]
NBS1 [52]	DSB sensing; ATM activation	Over-expressed in HNSCC tumors [71]
ADH5 [6]	Formaldehyde processing	Reduced expression in melanoma cells [72]

**Table 2:** ATM-deficiency in cancer.

Malignancy	ATM-deficient cell lines/number tested
T-cell prolymphocytic leukemia [73]	17/32
Mantle cell lymphoma [53]	12/28
Rhabdomyosarcoma [74]	7/17
Chronic lymphoblastic leukemia [54, 73]	16/50, 38/111
BRCA1-negative breast cancer [75]	12/36
BRCA2 negative breast cancer [75]	12/40
Acute lymphoblastic leukemia [54]	4/15
Non-BRCA1/BRCA2 negative breast cancers [75]	118/1106
Other lymphomas [53]	10/97
